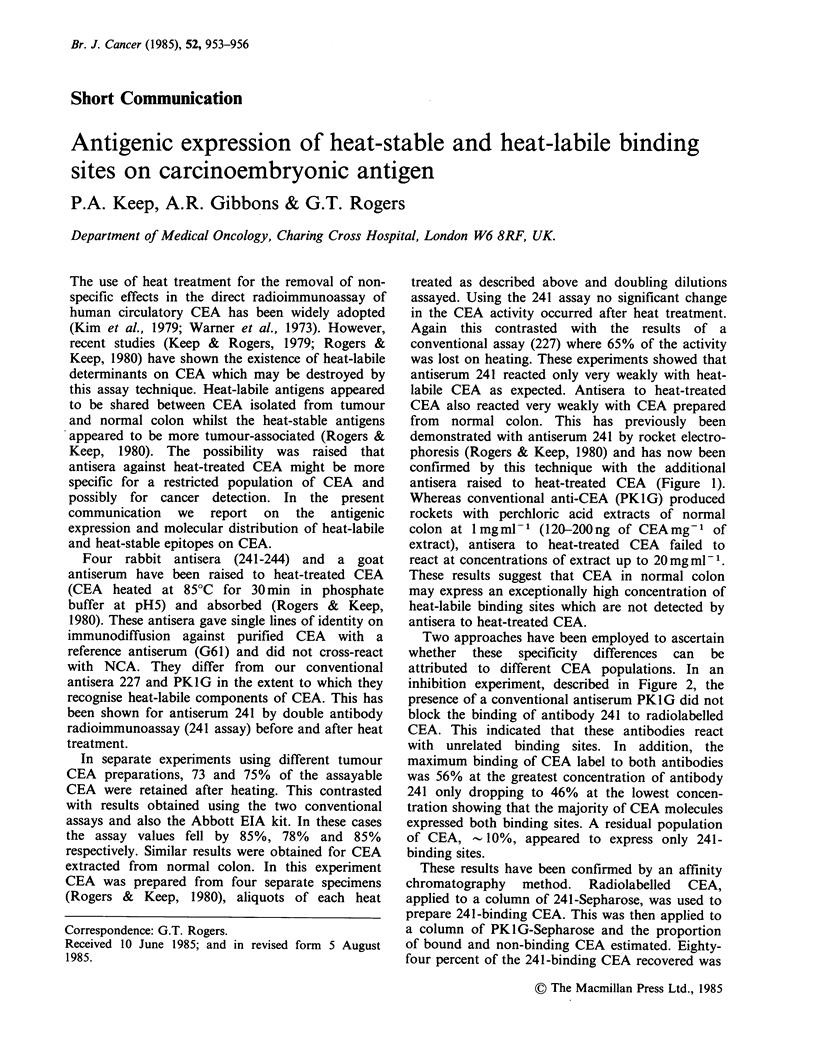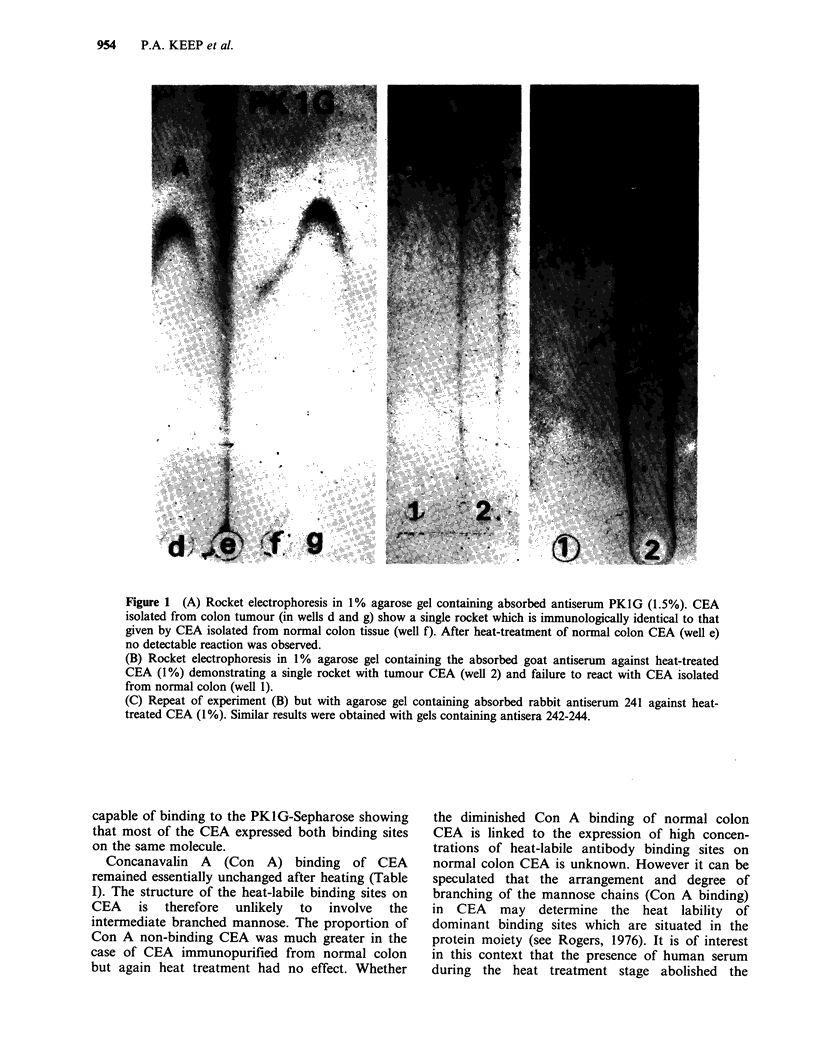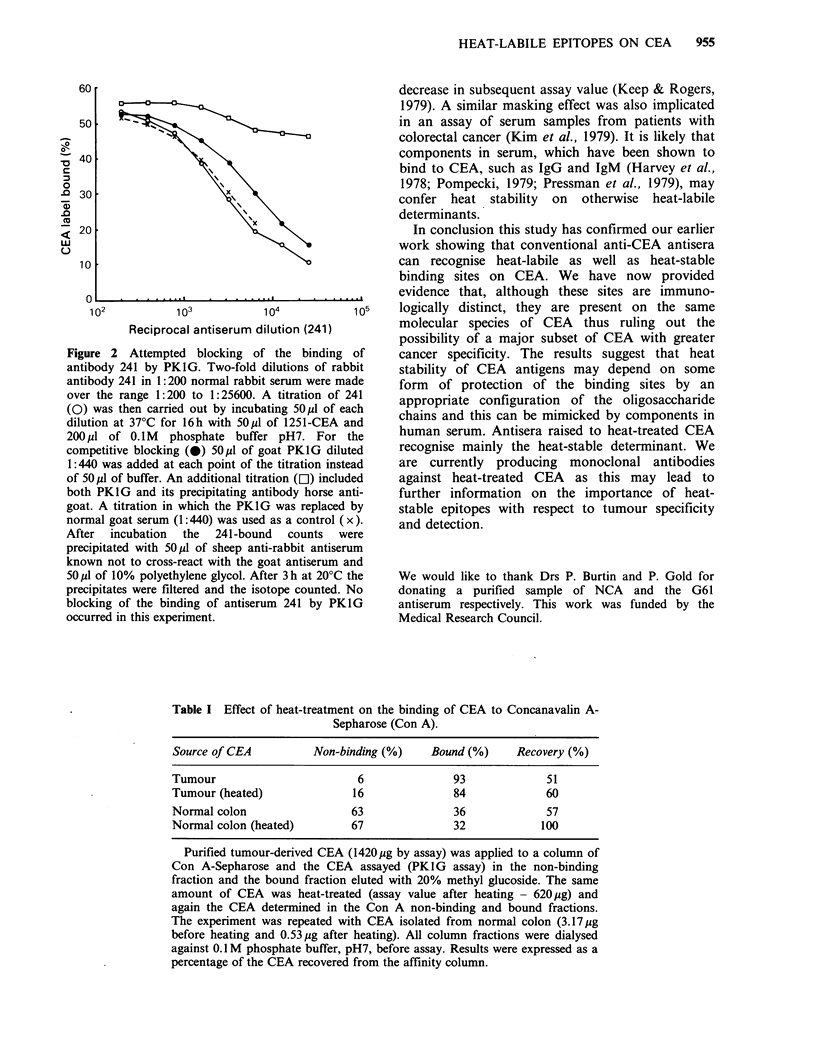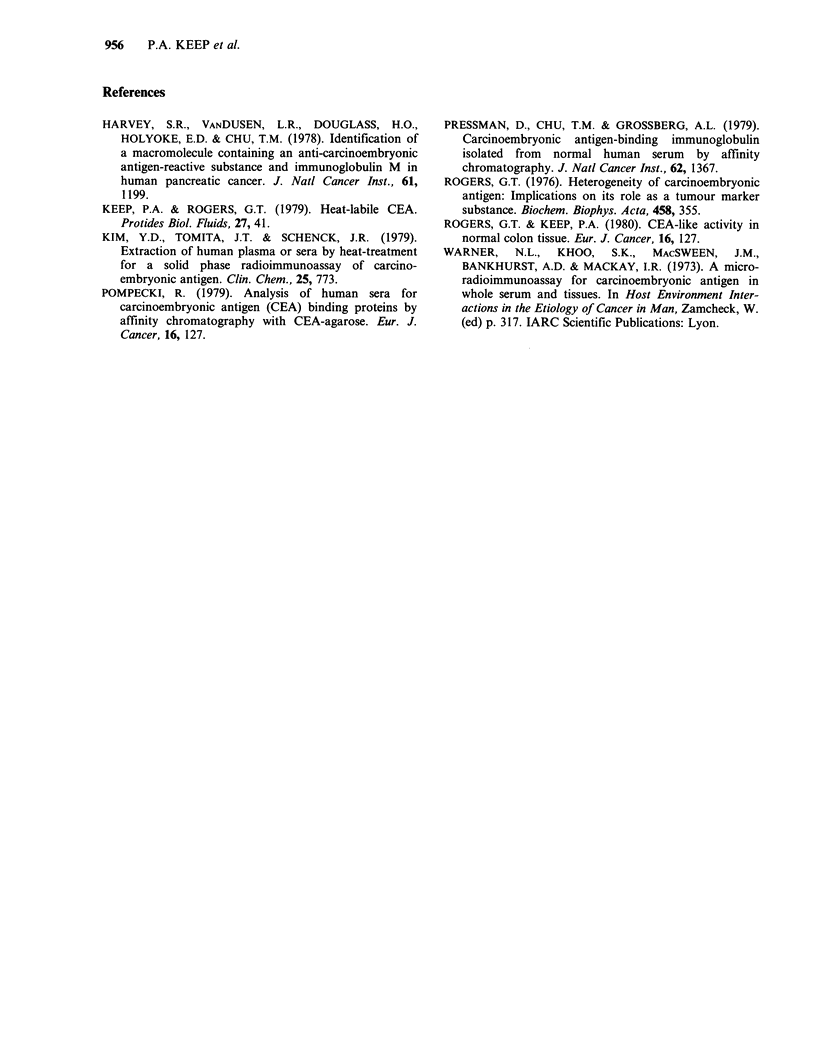# Antigenic expression of heat-stable and heat-labile binding sites on carcinoembryonic antigen.

**DOI:** 10.1038/bjc.1985.284

**Published:** 1985-12

**Authors:** P. A. Keep, A. R. Gibbons, G. T. Rogers

## Abstract

**Images:**


*Br. J. Cancer* (1985), **52**, 953–956

### Short Communication

### Antigenic expression of heat-stable and heat-labile binding sites on carcinoembryonic antigen

P.A. Keep, A.R. Gibbons & G.T. Rogers

*Department of Medical Oncology, Charing Cross Hospital, London W6 8RF, UK.*

The use of heat treatment for the removal of non-specific effects in the direct radioimmunoassay of human circulatory CEA has been widely adopted (Kim *et al.*, 1979; Warner *et al.*, 1973). However, recent studies (Keep & Rogers, 1979; Rogers & Keep, 1980) have shown the existence of heat-labile determinants on CEA which may be destroyed by this assay technique. Heat-labile antigens appeared to be shared between CEA isolated from tumour and normal colon whilst the heat-stable antigens appeared to be more tumour-associated (Rogers & Keep, 1980). The possibility was raised that antisera against heat-treated CEA might be more specific for a restricted population of CEA and possibly for cancer detection. In the present communication we report on the antigenic expression and molecular distribution of heat-labile and heat-stable epitopes on CEA.

Four rabbit antisera (241-244) and a goat antiserum have been raised to heat-treated CEA (CEA heated at 85°C for 30 min in phosphate buffer at pH5) and absorbed (Rogers & Keep, 1980). These antisera gave single lines of identity on immunodiffusion against purified CEA with a reference antiserum (G61) and did not cross-react with NCA. They differ from our conventional antisera 227 and PK1G in the extent to which they recognise heat-labile components of CEA. This has been shown for antiserum 241 by double antibody radioimmunoassay (241 assay) before and after heat treatment.

In separate experiments using different tumour CEA preparations, 73 and 75% of the assayable CEA were retained after heating. This contrasted with results obtained using the two conventional assays and also the Abbott EIA kit. In these cases the assay values fell by 85%, 78% and 85% respectively. Similar results were obtained for CEA extracted from normal colon. In this experiment CEA was prepared from four separate specimens (Rogers & Keep, 1980), aliquots of each heat treated as described above and doubling dilutions assayed. Using the 241 assay no significant change in the CEA activity occurred after heat treatment. Again this contrasted with the results of a conventional assay (227) where 65% of the activity was lost on heating. These experiments showed that antiserum 241 reacted only very weakly with heat-labile CEA as expected. Antisera to heat-treated CEA also reacted very weakly with CEA prepared from normal colon. This has previously been demonstrated with antiserum 241 by rocket electrophoresis (Rogers & Keep, 1980) and has now been confirmed by this technique with the additional antisera raised to heat-treated CEA (Figure 1). Whereas conventional anti-CEA (PK1G) produced rockets with perchloric acid extracts of normal colon at $1\,mg\,ml^{-1}$ (120–200 ng of CEA $mg^{-1}$ of extract), antisera to heat-treated CEA failed to react at concentrations of extract up to $20\,mg\,ml^{-1}$. These results suggest that CEA in normal colon may express an exceptionally high concentration of heat-labile binding sites which are not detected by antisera to heat-treated CEA.

Two approaches have been employed to ascertain whether these specificity differences can be attributed to different CEA populations. In an inhibition experiment, described in Figure 2, the presence of a conventional antiserum PK1G did not block the binding of antibody 241 to radiolabelled CEA. This indicated that these antibodies react with unrelated binding sites. In addition, the maximum binding of CEA label to both antibodies was 56% at the greatest concentration of antibody 241 only dropping to 46% at the lowest concentration showing that the majority of CEA molecules expressed both binding sites. A residual population of CEA, ~10%, appeared to express only 241-binding sites.

These results have been confirmed by an affinity chromatography method. Radiolabelled CEA, applied to a column of 241-Sepharose, was used to prepare 241-binding CEA. This was then applied to a column of PK1G-Sepharose and the proportion of bound and non-binding CEA estimated. Eighty-four percent of the 241-binding CEA recovered was

Correspondence: G.T. Rogers.
Received 10 June 1985; and in revised form 5 August 1985.

© The Macmillan Press Ltd., 1985

**Figure 1** (A) Rocket electrophoresis in 1% agarose gel containing absorbed antiserum PK1G (1.5%). CEA isolated from colon tumour (in wells d and g) show a single rocket which is immunologically identical to that given by CEA isolated from normal colon tissue (well f). After heat-treatment of normal colon CEA (well e) no detectable reaction was observed.
(B) Rocket electrophoresis in 1% agarose gel containing the absorbed goat antiserum against heat-treated CEA (1%) demonstrating a single rocket with tumour CEA (well 2) and failure to react with CEA isolated from normal colon (well 1).
(C) Repeat of experiment (B) but with agarose gel containing absorbed rabbit antiserum 241 against heat-treated CEA (1%). Similar results were obtained with gels containing antisera 242-244.

capable of binding to the PK1G-Sepharose showing that most of the CEA expressed both binding sites on the same molecule.

Concanavalin A (Con A) binding of CEA remained essentially unchanged after heating (Table I). The structure of the heat-labile binding sites on CEA is therefore unlikely to involve the intermediate branched mannose. The proportion of Con A non-binding CEA was much greater in the case of CEA immunopurified from normal colon but again heat treatment had no effect. Whether the diminished Con A binding of normal colon CEA is linked to the expression of high concentrations of heat-labile antibody binding sites on normal colon CEA is unknown. However it can be speculated that the arrangement and degree of branching of the mannose chains (Con A binding) in CEA may determine the heat lability of dominant binding sites which are situated in the protein moiety (see Rogers, 1976). It is of interest in this context that the presence of human serum during the heat treatment stage abolished the

**Figure 2** Attempted blocking of the binding of antibody 241 by PK1G. Two-fold dilutions of rabbit antibody 241 in 1:200 normal rabbit serum were made over the range 1:200 to 1:25600. A titration of 241 (○) was then carried out by incubating 50 μl of each dilution at 37°C for 16 h with 50 μl of 1251-CEA and 200 μl of 0.1M phosphate buffer pH7. For the competitive blocking (●) 50 μl of goat PK1G diluted 1:440 was added at each point of the titration instead of 50 μl of buffer. An additional titration (□) included both PK1G and its precipitating antibody horse anti-goat. A titration in which the PK1G was replaced by normal goat serum (1:440) was used as a control (×). After incubation the 241-bound counts were precipitated with 50 μl of sheep anti-rabbit antiserum known not to cross-react with the goat antiserum and 50 μl of 10% polyethylene glycol. After 3 h at 20°C the precipitates were filtered and the isotope counted. No blocking of the binding of antiserum 241 by PK1G occurred in this experiment.

decrease in subsequent assay value (Keep & Rogers, 1979). A similar masking effect was also implicated in an assay of serum samples from patients with colorectal cancer (Kim *et al.*, 1979). It is likely that components in serum, which have been shown to bind to CEA, such as IgG and IgM (Harvey *et al.*, 1978; Pompecki, 1979; Pressman *et al.*, 1979), may confer heat stability on otherwise heat-labile determinants.

In conclusion this study has confirmed our earlier work showing that conventional anti-CEA antisera can recognise heat-labile as well as heat-stable binding sites on CEA. We have now provided evidence that, although these sites are immunologically distinct, they are present on the same molecular species of CEA thus ruling out the possibility of a major subset of CEA with greater cancer specificity. The results suggest that heat stability of CEA antigens may depend on some form of protection of the binding sites by an appropriate configuration of the oligosaccharide chains and this can be mimicked by components in human serum. Antisera raised to heat-treated CEA recognise mainly the heat-stable determinant. We are currently producing monoclonal antibodies against heat-treated CEA as this may lead to further information on the importance of heat-stable epitopes with respect to tumour specificity and detection.

We would like to thank Drs P. Burtin and P. Gold for donating a purified sample of NCA and the G61 antiserum respectively. This work was funded by the Medical Research Council.

**Table I** Effect of heat-treatment on the binding of CEA to Concanavalin A-Sepharose (Con A).

| *Source of CEA* | *Non-binding* (%) | *Bound* (%) | *Recovery* (%) |
|---|---|---|---|
| Tumour | 6 | 93 | 51 |
| Tumour (heated) | 16 | 84 | 60 |
| Normal colon | 63 | 36 | 57 |
| Normal colon (heated) | 67 | 32 | 100 |

Purified tumour-derived CEA (1420 μg by assay) was applied to a column of Con A-Sepharose and the CEA assayed (PK1G assay) in the non-binding fraction and the bound fraction eluted with 20% methyl glucoside. The same amount of CEA was heat-treated (assay value after heating – 620 μg) and again the CEA determined in the Con A non-binding and bound fractions. The experiment was repeated with CEA isolated from normal colon (3.17 μg before heating and 0.53 μg after heating). All column fractions were dialysed against 0.1M phosphate buffer, pH7, before assay. Results were expressed as a percentage of the CEA recovered from the affinity column.

### References

HARVEY, S.R., VANDUSEN, L.R., DOUGLASS, H.O., HOLYOKE, E.D. & CHU, T.M. (1978). Identification of a macromolecule containing an anti-carcinoembryonic antigen-reactive substance and immunoglobulin M in human pancreatic cancer. *J. Natl Cancer Inst.*, **61**, 1199.

KEEP, P.A. & ROGERS, G.T. (1979). Heat-labile CEA. *Protides Biol. Fluids*, **27**, 41.

KIM, Y.D., TOMITA, J.T. & SCHENCK, J.R. (1979). Extraction of human plasma or sera by heat-treatment for a solid phase radioimmunoassay of carcino-embryonic antigen. *Clin. Chem.*, **25**, 773.

POMPECKI, R. (1979). Analysis of human sera for carcinoembryonic antigen (CEA) binding proteins by affinity chromatography with CEA-agarose. *Eur. J. Cancer*, **16**, 127.

PRESSMAN, D., CHU, T.M. & GROSSBERG, A.L. (1979). Carcinoembryonic antigen-binding immunoglobulin isolated from normal human serum by affinity chromatography. *J. Natl Cancer Inst.*, **62**, 1367.

ROGERS, G.T. (1976). Heterogeneity of carcinoembryonic antigen: Implications on its role as a tumour marker substance. *Biochem. Biophys. Acta*, **458**, 355.

ROGERS, G.T. & KEEP, P.A. (1980). CEA-like activity in normal colon tissue. *Eur. J. Cancer*, **16**, 127.

WARNER, N.L., KHOO, S.K., MACSWEEN, J.M., BANKHURST, A.D. & MACKAY, I.R. (1973). A micro-radioimmunoassay for carcinoembryonic antigen in whole serum and tissues. In *Host Environment Interactions in the Etiology of Cancer in Man*, Zamcheck, W. (ed) p. 317. IARC Scientific Publications: Lyon.